# Supplementary material for: Identification and Analysis of the Mechanism of Stem Mechanical Strength Enhancement for Maize Inbred Lines QY1
Source: Int J Mol Sci. 2024 Jul 27;25(15):8195. doi: 10.3390/ijms25158195 (PMC11312173; doi:10.3390/ijms25158195)
Supplement: Supplementary file 1 [file ijms-25-08195-s001.zip › ijms-3097079-supplementary.pdf]

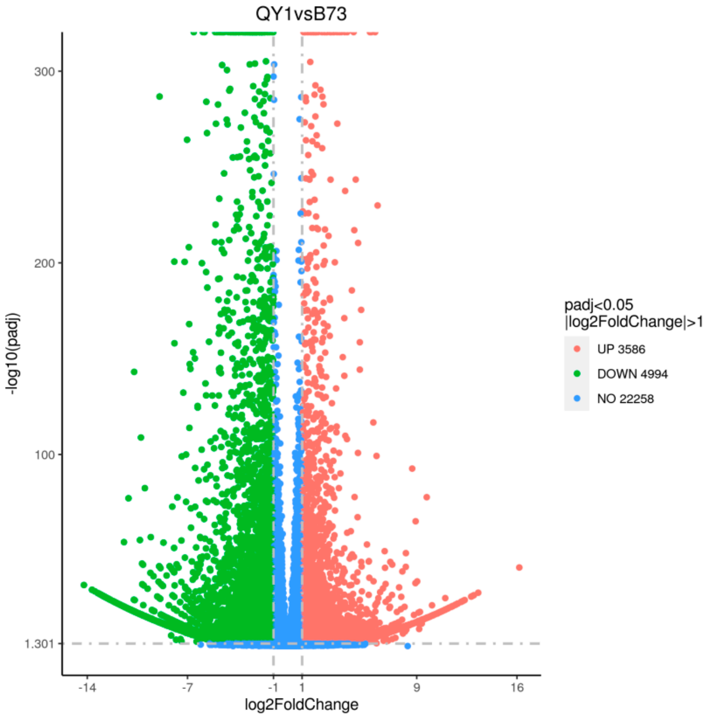

Supplementary Figure S1. QY1 vs B73 differential gene volcano map analysis

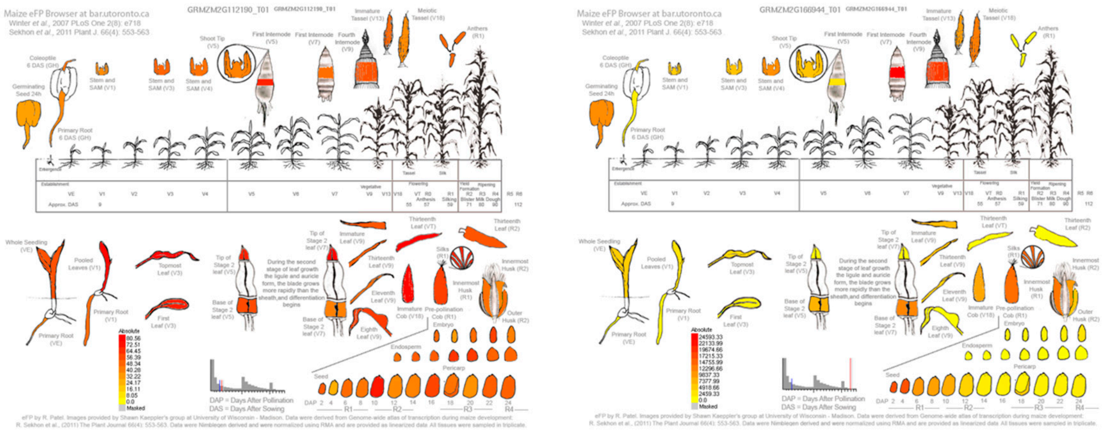

Zm00001eb187870 **Zm00001eb226490**

Supplementary Figure S2. *Zm00001eb187870*, *Zm00001eb226490* gene expression pattern map

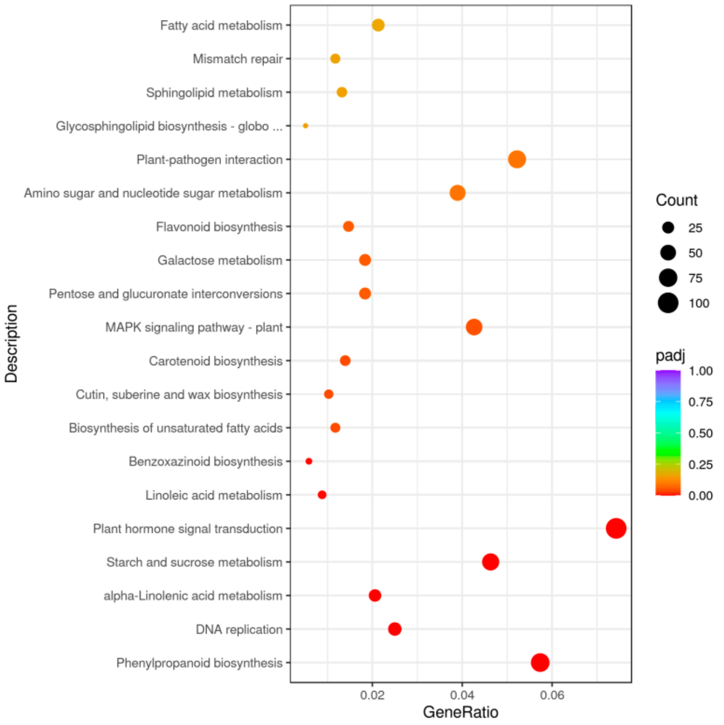

Supplementary Figure S3. KEGG analysis of QY1 vs B73 differential genes

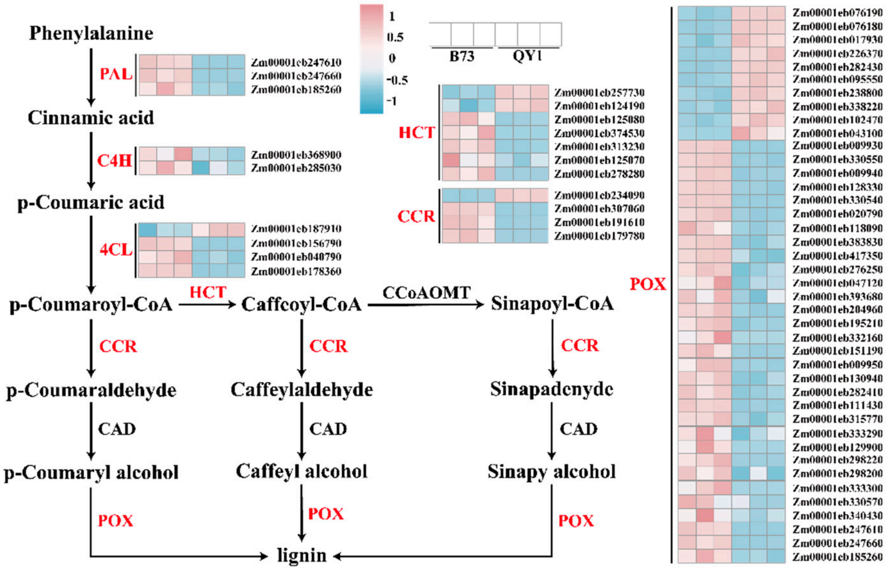

Supplementary Figure S4. Heatmap analysis of QY1 vs B73 phenylpropane biosynthesis metabolic pathways and differential genes

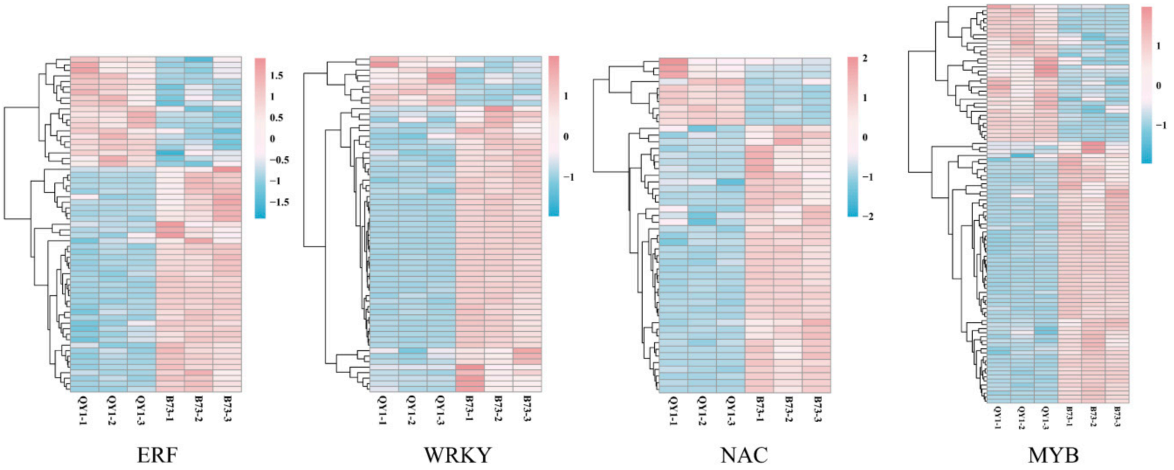

Supplementary Figure S5. Heatmap analysis of QY1 vs B73 transcription factor differential genes

Supplementary Table S1. Forward (F)/reverse (R) primer sequences used for qRT-PCR.

Gene Number	Primer Sequence
Zm00001eb226490	F:TGATGTTTGGCTGGACGTGA<br>R:CAGTGACGCTGACTGACCAT
Zm00001eb330500	F:GACGATCGACAACGGCCTAA<br>R:AGCAGCTATCAGTGCGATGC
Zm00001eb112510	F:TGTGAGGTGAATGTCTCGCC<br>R:AGCAATGCAAAGCAACCCAG
Zm00001eb353540	F:CACATCGCTGCAGGTTTTCA<br>R:CTAGGCTTGCTCGGCTGTAA
Zm00001eb411910	F:TTCCGACTCACAAAGAGCCC<br>R:GCGCACATGGATCCGTAGAA
Zm00001eb293700	F:GTAAACAGCTTCTCGACCGC<br>R:AGCGGCCCGTGGTATAATTC
Zm00001eb414360	F:CCTTTGGATACAGGCATGCAG<br>R:ACATGTACGTAGCTAGTTCGG
Zm00001eb203620	F:GGGACCGTGTCAACACTCAT<br>R:TGGAAGGTGTATGGTTGCCC
Actin	F:TACGAGATGCCTGATGGTCAGGTCA<br>R:TGGAGTTGTACGTGGCCTCATGGAC

Supplementary Table S2. number of differential genes in the QY1 vs B73 KEGG-enriched pathway

Description	Count	Up	Down
Plant hormone signal transduction	101	34	67
Phenylpropanoid biosynthesis	78	28	50
Starch and sucrose metabolism	63	40	23
MAPK signaling pathway - plant	58	24	34
DNA replication	34	30	4
alpha-Linolenic acid metabolism	28	15	13
Carotenoid biosynthesis	19	10	9
Biosynthesis of unsaturated fatty acids	16	11	5
Cutin, suberine and wax biosynthesis	14	4	10
Linoleic acid metabolism	12	4	8
Benzoxazinoid biosynthesis	8	0	8

Supplementary Table S3. QY1 vs B73 transcription factor-related differential genes

Gene Family	Gene Number	Gene Annotation	log_2 Fold Change
WRKY	*Zm00001eb322330*	*WRKY129* - WRKY-transcription factor 129	6.448243428
WRKY	*Zm00001eb163850*	*WRKY4* - WRKY-transcription factor 4	4.819137722
WRKY	*Zm00001eb020400*	*WRKY75* - WRKY-transcription factor 75	4.348871624
WRKY	*Zm00001eb237820*	*WRKY2* - WRKY-transcription factor 2	1.683798937
WRKY	*Zm00001eb359470*	*WRKY86* - WRKY-transcription factor 86	-10.15333208
ERF	*Zm00001eb062200*	*EREBP159* - AP2-EREBP-transcription factor 159	7.997857419
ERF	*Zm00001eb244060*	*EREBP157* - AP2-EREBP-transcription factor 157	3.502808341
ERF	*Zm00001eb202570*	*EREBP111* - AP2-EREBP-transcription factor 111	2.91464162
ERF	*Zm00001eb408830*	*EREBP218* - AP2-EREBP-transcription factor 218	-7.222380928
NAC	*Zm00001eb139220*	*NAC100* - NAC-transcription factor 100	5.084482205
NAC	*Zm00001eb324550*	*NAC132* - NAC-transcription factor 132	4.749533144
NAC	*Zm00001eb264380*	*NAC119* - NAC-transcription factor 119	3.983285245
NAC	*Zm00001eb015630*	*NAC44* - NAC-transcription factor 44	2.98525746
NAC	*Zm00001eb032650*	*NAC50* - NAC-transcription factor 50	-5.943533372
NAC	*Zm00001eb334160*	*NAC118* - NAC-transcription factor 118	-6.358257771
MYB	*Zm00001eb366790*	*MYB55* - MYB-related-transcription factor 55	5.077079455
MYB	*Zm00001eb383720*	*GLK2* - G2-like-transcription factor 2	5.003175438
MYB	*Zm00001eb213800*	*MYB12* - MYB-transcription factor 12	-8.522703255
MYB	*Zm00001eb041330*	*MYB33*- MYB-transcription factor 33	-8.585904994
MYB	*Zm00001eb010890*	umc2397	-11.89286717
